# Pharyngeal oxygen administration increases the time to serious desaturation at intubation in acute lung injury: an experimental study

**DOI:** 10.1186/cc9027

**Published:** 2010-05-24

**Authors:** Joakim Engström, Göran Hedenstierna, Anders Larsson

**Affiliations:** 1 Department of Anesthesiology and Intensive Care, Uppsala University, ANIVA ing 70 1tr, University hospital, S 75185 Uppsala, Sweden; 2 Department of Clinical Physiology, Uppsala University, Ing 30, 4 tr, University hospital, S-75185 Uppsala, Sweden; 3 Department of Anesthesiology and Intensive Care, Uppsala University, ANIVA ing 70 1tr, University hospital, S 75185 Uppsala, Sweden

## Abstract

**Introduction:**

Endotracheal intubation in critically ill patients is associated with severe life-threatening complications in about 20%, mainly due to hypoxemia. We hypothesized that apneic oxygenation via a pharyngeal catheter during the endotracheal intubation procedure would prevent or increase the time to life-threatening hypoxemia and tested this hypothesis in an acute lung injury animal model.

**Methods:**

Eight anesthetized piglets with collapse-prone lungs induced by lung lavage were ventilated with a fraction of inspired oxygen of 1.0 and a positive end-expiratory pressure of 5 cmH_2_O. The shunt fraction was calculated after obtaining arterial and mixed venous blood gases. The trachea was extubated, and in randomized order each animal received either 10 L oxygen per minute or no oxygen via a pharyngeal catheter, and the time to desaturation to pulse oximeter saturation (SpO_2_) 60% was measured. If SpO_2 _was maintained at over 60%, the experiment ended when 10 minutes had elapsed.

**Results:**

Without pharyngeal oxygen, the animals desaturated after 103 (88-111) seconds (median and interquartile range), whereas with pharyngeal oxygen five animals had a SpO_2 _> 60% for the 10-minute experimental period, one animal desaturated after 7 minutes, and two animals desaturated within 90 seconds (*P *< 0.016, Wilcoxon signed rank test). The time to desaturation was related to shunt fraction (R^2 ^= 0.81, *P *= 0.002, linear regression); the animals that desaturated within 90 seconds had shunt fractions >40%, whereas the others had shunt fractions <25%.

**Conclusions:**

In this experimental acute lung injury model, pharyngeal oxygen administration markedly prolonged the time to severe desaturation during apnea, suggesting that this technique might be useful when intubating critically ill patients with acute respiratory failure.

## Introduction

Endotracheal intubation is one of the most hazardous procedures in the ICU. This is because the patients are usually in a compromised circulatory and pulmonary condition in which low functional residual capacity in combination with a pulmonary shunt and increased oxygen consumption contribute to rapidly developing hypoxemia during apnea [1-4]. Although complications may be reduced by rigorously following protocols, more than 20% of endotracheal intubations in patients in the ICU are associated with serious complications, usually caused by severe hypoxemia [5]. Furthermore, in more than 10% of patients more than two intubation attempts are made, and in 10% the intubation procedure takes more than 10 minutes [3,4]. Therefore, it is important to extend the period of adequate oxygenation during the apneic period needed for the intubation for as long as possible. The routine way to do this is by preoxygenation via a nose-mouth mask [6,7]. However, this technique is not always effective in patients with respiratory distress [8,9]. Other techniques have therefore been proposed to reduce the risk of hypoxemia-like non-invasive ventilation with positive end-expiratory pressure (PEEP) during preoxygenation [10-12]. Although this technique has been found to be useful and has improved oxygenation under and after intubation, the lungs may collapse within seconds after removal of the positive pressure. Therefore, theoretically, this technique may not be effective in patients with acute respiratory distress syndrome [13].

Apneic oxygenation, that is, delivering 100% oxygen to the airways and lungs without ventilation, can maintain adequate oxygenation for long periods in patients with normal lungs, in intensive care patients in connection with the diagnosis of brain death, and in experimental animals [14-17]. In addition, apneic oxygenation has been shown to prolong the time to hypoxemia in patients with healthy lungs and during intubation of obese patients in connection with anesthesia [18-20]. However, this technique has not been reported to be used in acute hypoxic respiratory failure in either patients or in experimental lung injury. Furthermore, it is not known whether the technique is effective if intrapulmonary shunt fractions are high. We hypothesized that pharyngeal oxygen administration would prevent or increase the time to life-threatening hypoxemia at intubation procedures during apnea in conditions with collapse-prone lungs with high shunt fractions. The aim of the study was to test this hypothesis in an experimental large-animal model of acute lung injury using different intrapulmonary shunt fractions.

This article reports that pharyngeal apneic oxygenation prevented or prolonged the time to life-threatening hypoxemia during a simulated intubation procedure in an animal model of acute lung injury.

## Materials and methods

The study was approved by the Animal Research Ethics Committee at Uppsala University, Sweden, and the National Institute of Health guidelines for animal research were followed.

### Anesthesia, ventilation, instrumentation, and monitoring

Eight pigs (weighing 23 to 28 kg) were premedicated with Zoletil Forte (tiletamine and zolazepam (Virbac Laboratories, Carros, France) 6 mg kg^-1 ^and Rompun (xylazine hydrocloride, Bayer Animal Health, Lyngby, Denmark) 2.2 mg kg^-1 ^intramuscularly. After 5 to 10 minutes the pig was placed supine on a table, the trachea was intubated with a 7 mm ID endotracheal tube (Mallinckrodt Medical, Athlone, Ireland), and the lungs were ventilated in a volume-control mode by a Servo-I (Maquet, Solna, Sweden) with tidal volume (VT) of 8 mL kg^-1^, fraction of inspired oxygen (FiO_2_) of 0.5, and PEEP of 5 cmH_2_O. The rate was adjusted to keep end-tidal carbon dioxide tension at 5 to 6 kPa (Siemens SC 9000XL, Dräger, Germany). Just after the endotracheal intubation, a bolus of fentanyl 0.02 mg kg^-1 ^was given intravenously. Anesthesia was then maintained with ketamine 30 mg kg^-1^h^-1 ^and midazolam 0.1 mg kg^-1 ^h^-1^. The depth of the anesthesia was tested intermittently with pain stimulation of the front toes. If the anesthesia was deemed insufficient, fentanyl 0.2 mg was given intravenously. During the first two hours, 10 ml kg^-1^h^-1 ^Ringer's acetate was infused intravenously, and then the infusion rate was altered to 5 ml kg^-1 ^h^-1 ^intravenously. After open dissection of the neck vessels, an arterial catheter was inserted into the right carotid artery for blood gas sampling and blood pressure monitoring, and a central venous catheter was inserted via the right external jugular vein. In addition, a pulmonary arterial catheter (Criti Cath No7; Ohmeda Pte Ltd, Singapore) for measurement of cardiac output and pulmonary artery pressure was introduced via the right external jugular vein, and the position in the pulmonary artery was assured by pressure monitoring. Cardiac output was obtained as the mean of three values measured by thermodilution after injection of 10 mL ice-cold saline into the central venous catheter (Siemens SC 9000XL, Dräger, Germany). A bladder catheter was inserted suprapubically to monitor urine production. Electrocardiographic monitoring was started, and pulse oximeter (Siemens SC 9000XL, Dräger, Germany) oxygen saturation (SpO_2_) was measured at the base of the tail.

### Calculation of venous admixture (shunt) and compliance of the respiratory system

Venous admixture was calculated using the standard formula [21]. A FiO_2 _of 1.0 was used during sampling of blood gases, so we regard our reported values for the venous admixture to be a very close estimate of the intrapulmonary shunt [21].

Compliance of the respiratory system (Crs) was calculated as: VT/(EIP-PEEP), where EIP is the end-inspiratory plateau pressure. Both EIP and PEEP were measured after a 15-second pause.

### Experimental protocol

**Figure 1:**
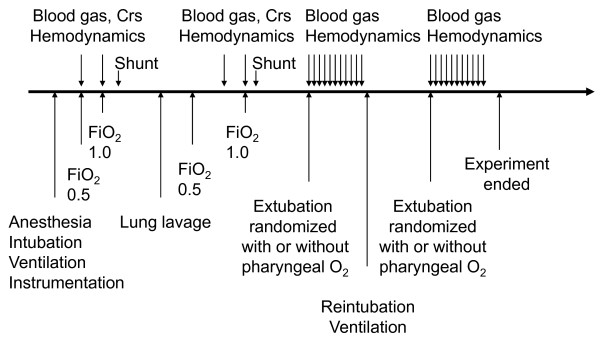
**Outline of the experiment**. The arrows above the horizontal line indicate measurements, whereas the arrows below the line indicate interventions. The two periods were randomized during which pharyngeal oxygen was or was not administered. Crs, compliance of the respiratory system; FiO2, fraction of inspired oxygen.

The outline of the study is given in Figure 1. After the instrumentation, arterial blood was sampled for measurement of oxygen tension, carbon dioxide tension, pH, base excess (ABL 3, Radiometer, Copenhagen, Denmark), and oxygen hemoglobin saturation (OSM 3, Radiometer, Copenhagen, Denmark). Thereafter, FiO_2 _was changed to 1.0 and after a further five minutes, arterial and mixed venous blood gases were obtained for calculation of the pulmonary shunt. In addition, Crs, cardiac output, heart rate, and systemic and pulmonary pressures were registered.

Thereafter, a collapse-prone lung was created by lung lavage. Before the lavage procedure, the animals received fentanyl 0.2 mg and pancuronium 3 mg intravenously. To achieve different levels of lung collapse and shunt fraction, the lungs were lavaged 3 to 10 times with 20 mL/kg isotonic saline at 38°C. FiO_2 _was reduced to 0.5, and the animals were left undisturbed for 30 minutes. If SpO_2 _decreased below 85%, FiO_2 _was increased to achieve a SpO_2 _above 85%. After 30 minutes, a new arterial blood gas sample was taken.

A 12 French catheter was placed via one nostril (or if not possible, via the mouth) with its distal opening in the pharynx. FiO_2 _was changed to 1.0. After five minutes, arterial and mixed venous blood samples were taken for shunt calculation, and hemodynamic data and Crs were registered. Fentanyl 0.2 mg and pancuronium 6 mg were given intravenously to assure that no attempts at spontaneous breathing occurred. In randomized order, either oxygen 10 L per minute or no oxygen (no flow) was delivered via the pharyngeal catheter. The endotracheal tube was removed after the larynx had been localized by a laryngoscope, and the time was registered at which the SpO_2 _had fallen to 60%. After tracheal extubation, the laryngoscope was maintained in place.

Arterial blood gases were sampled before the tracheal extubation and then every minute until and when SpO_2 _was below 60% or until 10 minutes had elapsed. At similar time points, heart rate and systemic and pulmonary pressures were registered.

The trachea was again intubated; the lungs were ventilated with unchanged ventilator settings, except that the respiratory rate was increased in order to normalize end-tidal carbon dioxide. When end-tidal carbon dioxide was normalized, the lungs were ventilated for five minutes at the same rate as before the extubation. The trachea was again extubated and the not-studied preoxygenation technique (without or with pharyngeal oxygen) was examined in the same way as described previously. Thereafter, the experiment ended, and the animal was euthanized by an overdose of potassium chloride given intravenously. No animal died before the completion of the experiment.

### Statistics

For *P *values of 0.05 and a power of 0.8 for the primary outcome variable, time to life-threatening hypoxemia (SpO_2 _<60%), eight animals were considered sufficient. For analyses of the differences between the preoxygenation techniques, Wilcoxon signed-rank test was used. Linear regression was used to analyze the relation between time to life-threatening hypoxemia and shunt fraction. The data are reported as medians with interquartile ranges unless otherwise indicated.

For the statistical analyses, the Sigmastat statistical program (Systat, Software Inc, Point Richmond, CA, USA) was used. *P *less than 0.05 was considered as statistically significant.

## Results

### Effect of lung lavage

The partial pressure of arterial oxygen (PaO_2_) on FiO_2 _0.5 and 1.0 decreased from 33 (31 to 35) to 13 (8 to 16) kPa (*P *= 0.008), and 71 (68 to 75) to 47 (21 to 52) kPa (*P *= 0.008), respectively. Crs decreased from 25 (23 to 27) to 9 (8 to 10) mL cmH2O^-1 ^(*P *= 0.008). Venous admixture with FiO_2 _1.0 (shunt fraction) increased from 7% (5 to 8%) to 19% (13 to 35%; *P *= 0. 008) with, as planned, a wide range (9 to 54%).

### Time to life-threatening hypoxemia

**Figure 2:**
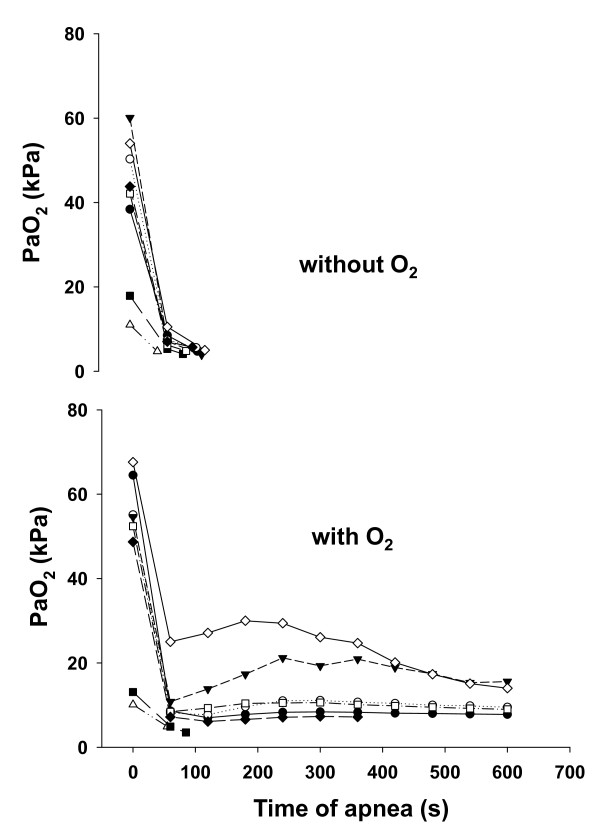
**Arterial oxygen tension (PaO_2_) versus time of apnea without (upper panel) and with (lower panel) pharyngeal oxygen administration**. The symbols and lines depict the individual values.

Without pharyngeal oxygen, the time to SpO_2 _below 60% was 103 (88 to 111) seconds, and with pharyngeal oxygen, three animals desaturated (after 55 seconds, 85 seconds, and 7 minutes), whereas the other five animals had adequate oxygenation during the whole 10-minute study period (*P *= 0.016). The individual PaO_2 _values at the different time points are shown in Figure 2.

### Relation between shunt fraction and time to life-threatening hypoxemia with pharyngeal oxygen

**Figure 3:**
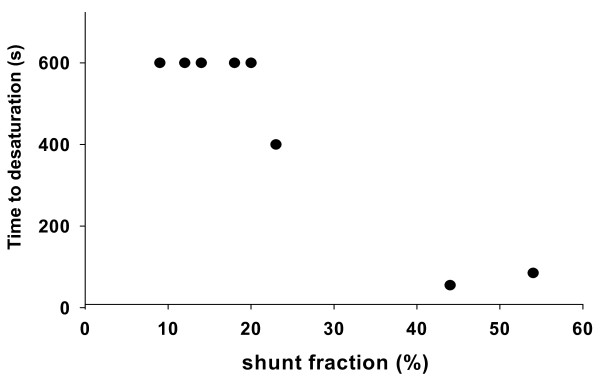
**Time to desaturation below 60% as estimated by pulse oximetry versus shunt fraction on pharyngeal oxygen administration**. The dots depict the individual values.

There is a close correlation between shunt and time to desaturation (Figure 3). If 600 seconds are used in the equation for the animals that did not desaturate during the study period, the equation is: Time (seconds) = 937 - 8.5 × shunt (%) (R^2 ^= 0.81, *P *= 0.002). When the shunt was less than 20%, no desaturation occurred during the 10-minute time frame, but when shunt was above 44%, desaturation occurred within 90 seconds.

### Carbon dioxide and pH during apnea

During the 10-minute apnea period with pharyngeal oxygen, partial pressure of arterial carbon dioxide (PaCO_2_) increased from 6.4 (6.2 to 7.0) to 17.1 (16.3 to 17.3) kPa (*P *< 0.05) and pH decreased from 7.36 (7.34 to 7.38) to 7.03 (7.02 to 7.05; *P *< 0.05).

### Hemodynamics

Lung lavage did not affect hemodynamics significantly, whereas prolonged apnea was associated with an increase in heart rate from 78 (65 to 92) to 102 (87 to 109) beats per minute (*P *= 0.023), mean arterial pressure from 80 (70 to 91) to 94 (84 to 93) mmHg (*P *= 0.03), and mean pulmonary arterial pressure from 22 (18 to 25) to 33 (28 to 39) mmHg (*P *= 0.004).

## Discussion

This porcine study showed that when the shunt fraction was below about 25% on a PEEP of 5 cmH_2_O, pharyngeal oxygen administration given during apnea in connection with simulated endotracheal intubation either prevented life-threatening hypoxemia or substantially increased the time until it took place.

Endotracheal intubation in intensive care patients is associated with severe complications, many of which are caused by hypoxemia during the apneic period [1-4]. Furthermore, severe hypoxemia is common in connection with endotracheal intubation in the emergency department or in prehospital care [1,2,22]. We hypothesized that hypoxemia could be ameliorated or prevented by apneic oxygenation, achieved by administering oxygen via a pharyngeal catheter during preoxygenation and during the intubation attempt. The technique of apneic oxygenation is, however, not new. It was first described by Draper and Whitehead 1944 in apneic dogs [14]. Enghoff and colleagues described as early as 1951 that this technique was able to oxygenate a volunteer for a prolonged time [15]. They and others later elaborated the technique in animal experiments as well as in patients and showed that the technique could maintain adequate oxygenation for up to 30 minutes [15-17]. Oxygen is absorbed at a rate of about 250 mL per minute in a healthy, resting, normal-weight adult subject. This creates a force that sucks gas into the alveoli. If oxygen is administered, it replaces the consumed alveolar oxygen [16]. Alveolar carbon dioxide does not increase more than about 0.5 kPa per minute because the carbon dioxide in the blood is buffered by the erythrocytes and dissociated in the tissue. Therefore alveolar oxygen concentration remains high for a prolonged period [16].

Apneic oxygenation is, however, seldom used nowadays, except in connection with the diagnosis of brain death. Teller and colleagues showed in 1988 that before intubation in anesthetized patients with healthy lungs, the technique could maintain excellent oxygenation for at least 10 minutes and proposed that 'this technique may be beneficial in situations when extra minutes are needed to gain control of the airway' [18]. Apneic oxygenation has been shown to be effective in connection with endotracheal intubation in obese patients undergoing anesthesia, but it has not been included as a strong recommendation in the American Society of Anesthesiologist's difficult airway algorithm, and, furthermore, there are no reports of its use in critically ill patients or in acute respiratory failure models [20,23].

We wanted to examine the difference in time to life-threatening apnea in subjects with collapse-prone lungs between the, at present, best available technique of preoxygenation, that is, ventilation with 100% oxygen with PEEP [11], with a combination of this technique and apneic oxygenation via pharyngeal oxygen administration. This cannot be performed in humans and therefore we used a porcine model. We showed that apneic oxygenation increased the time to life-threatening hypoxemia when the shunt was less than 25%. At shunt levels above 40%, only a minor increase was seen, about 10 seconds. However, the shunt was calculated with a PEEP of 5 cmH_2_O, and not with zero end-expiratory pressure, and it is clear from Figure 2 that PaO_2 _decreased markedly, even during pharyngeal oxygen administration, by one minute after the trachea was extubated and the airway left open to atmospheric pressure. Nielsen and colleagues have previously found in pigs with healthy lungs that apneic oxygenation at zero end-expiratory pressure could maintain a high PaO_2 _for at least 10 minutes [24]. Furthermore, the same group has shown that pigs with collapse-prone lungs could be well oxygenated for at least seven hours by apneic oxygenation with 20 cmH_2_O of continuous positive airway pressure combined extracorporeal carbon dioxide removal [25]. Therefore, we assume that the initial drop in PaO_2 _seen in the present study was caused by a rapid collapse of lung regions induced by the removal of PEEP. Indeed, in pigs with lavage-injured lungs, the time constant for lung collapse is about 16 seconds [26]. For these reasons, we believe that the PaO_2 _at one minute is a representative PaO_2 _of the shunt at zero end-expiratory pressure. If the PaO_2 _values measured at one minute in our study are inserted in Nunn's shunt diagram, the shunts are, in fact, 10% higher than what we found [21]. Thus, we believe that pharyngeal oxygen might be effective at shunt fractions up to 40%, which is the upper shunt fraction at which, according to Nunn, oxygen administration should improve arterial oxygenation [21].

It is obvious that PaCO_2 _increases during prolonged apnea. In our study the increase was most pronounced during the first minutes, and thereafter it was 0.5 to 1 kPa per minute. After 10 minutes, PaCO_2 _was about 17 kPa and pH had decreased to 7.0. However, this degree of hypercapnic acidosis did not markedly compromise circulation; heart rate and arterial pressure increased probably due to increased sympathetic activity, which also could have contributed to the increased pulmonary artery pressure. On the other hand, the successive increase in alveolar carbon dioxide diluting and reducing alveolar oxygen could be a reason why the pig with a moderate shunt did not tolerate more than seven minutes of apnea.

We believe that the major reason why the technique of apneic oxygenation via a pharyngeal catheter is seldom used in clinical practice is that it is not well known. However, it could also be due to its potential drawbacks. Firstly, application of the catheter might be cumbersome for the patient. However, we believe this is a minor problem, and furthermore, high flow oxygen can probably be administered in short catheters via the nostrils if no severe nasal obstruction exists. Secondly, the catheter can by mistake be inserted into the pharyngeal submucosa or into the esophagus. These errors should, however, be easily recognized. Thirdly, a high concentration of oxygen might cause absorption atelectasis and increase the pulmonary shunt [27]. However, absorption atelectasis is easily treated by a lung recruitment maneuver after a successful intubation [28].

Our study has several limitations. Firstly, it was carried out in pigs. Secondly, their body weights were about 25 kg. Thus, the ratio between end-expiratory lung volume (oxygen depot) and oxygen consumption is low, making the apneic period before desaturation shorter than in adult patients. Thirdly, lung lavage does not cause acute respiratory failure as found in patients; however, it induces a lung that rapidly collapses after removal of the positive airway pressure, and therefore we believe the model was adequate for this purpose. Fourthly, we used a limited number of pigs, so we did not catch the full spectrum of pulmonary shunt fractions, and we measured shunts only with 5 cmH_2_O of PEEP and not with zero end-expiratory pressure. Thus, we did not determine the exact upper limit of the shunt fraction at which pharyngeal oxygen is effective. Finally, we did not examine the effect of pharyngeal oxygen in conditions with severe upper airway obstruction. However, if 250 mL gas per minute can pass through an obstruction, apneic oxygenation should be useful.

## Conclusions

This porcine study showed that pharyngeal oxygen administration during apnea at an intubation procedure prevented or considerably increased the time to life-threatening hypoxemia at shunt fractions at least up to 25%. This technique might be implemented in airway algorithms for the intubation of hypoxemic patients, for example, in the ICU, in the emergency room, or in prehospital care or of patients with difficult airways.

## Key messages

• Pharyngeal oxygen administration prevents or delays hypoxemia during apnea in connection with tracheal intubation in an acute lung injury model.

• Pharyngeal oxygen administration might therefore be considered at tracheal intubation in critically ill patients.

## Abbreviations

Crs: compliance of the respiratory system; EIP: end-inspiratory plateau pressure; FiO_2_: fraction of inspired oxygen; PaCO_2_: partial pressure of arterial carbon dioxide; PaO_2_: partial pressure of arterial oxygen; PEEP: positive end-expiratory pressure; SpO_2_: pulse oximeter oxygen saturation; VT: tidal volume.

## Competing interests

The authors declare that they have no competing interests.

## Authors' contributions

JB participated in the design and acquisition of data, as well as helping to draft the manuscript. GH participated in the design of the study and in the revision of the manuscript. AL conceived the study, participated in the design and the data acquisition, performed the statistical analysis, and drafted the manuscript. All authors read and approved the final manuscript.

## References

[B1] Schwartz DE Matthay MA Cohen NE Death and other complications of emergency airway management in critical adults: A prospective investigation of 297 tracheal intubations Anesthesiology 1995 82 367 376 10.1097/00000542-199502000-00007 7856895

[B2] Mort TC Emergency tracheal intubation: Complications associated with repeated laryngoscopic attempts Anesth Analg 2004 99 607 613 10.1213/01.ANE.0000122825.04923.15 15271750

[B3] Griesdale DEG Bosma TL Kurth T Isac G Chittock DR Complications of endotracheal intubation in the critically ill Intensive Care Med 2008 34 1835 1842 10.1007/s00134-008-1205-6 18604519

[B4] Jaber S Amraoui J Lefrant J-Y Arich C Cohendy R Landreau L Calvet Y Capdevila X Mahamat A Eledjam JJ Clinical practice and risk factors for immediate complications of endotracheal intubation in the intensive care unit: A prospective, multiple-center study Crit Care Med 2006 34 2355 2361 10.1097/01.CCM.0000233879.58720.87 16850003

[B5] Jaber S Jung B Corne P Sebbane M Muller L Chanques G Verzilli D Jonquet O Eledjam J-J Lefrant J-Y An intervention to decrease complications related to endotracheal intubation in the intensive care unit: a prospective, multiple-center study Intensive Care Med 2010 36 248 255 10.1007/s00134-009-1717-8 19921148

[B6] Benumof JL Preoxygenation. Best method for both efficacy and efficiency? Anesthesiology 1999 91 603 605 10.1097/00000542-199909000-00006 10485765

[B7] Reynolds SF Heffner J Airway management of the critically ill patient Chest 2005 127 1397 1412 10.1378/chest.127.4.1397 15821222

[B8] Mort TC Preoxygenation in critically ill patients requiring emergency tracheal intubation Crit Care Med 2005 33 2672 2675 10.1097/01.CCM.0000187131.67594.9E 16276196

[B9] Mort TC Waberski BH Clive J Extending the preoxygenation period from 4 to 8 mins in critically ill patients undergoing emergency intubation Crit Care Med 2009 37 68 71 10.1097/CCM.0b013e318192845e 19050620

[B10] El-Khatib MF Kanazi G Baraka AS Noninvasive bilevel positive airway pressure for preoxygenation of the critically ill morbidly obese patient Can J Anesth 2007 54 744 747 10.1007/BF03026871 17766742

[B11] Delay JM Sebbane M Jung B Nocca D Verzilli D Pouzeratte Y Kamel ME Fabre JM Eledjam JJ Saber S The effectiveness of noninvasive positive pressure ventilation to enhance preoxygenation in morbidly obese patients: a randomized controlled study Anesth Analg 2008 107 1707 1713 10.1213/ane.0b013e318183909b 18931236

[B12] Baillard C Fosse J-P Sebbane M Chanques G Vincent F Courouble P Cohen Y Eledjam JJ Adnet F Jaber S Noninvasive ventilation improves preoxygenation before intubation of hypoxic patients Am J Respir Crit Care Med 2006 174 171 177 10.1164/rccm.200509-1507OC 16627862

[B13] Dyhr T Bonde J Larsson A Lung recruitment manoeuvres are effective in regaining lung volume and oxygenation after open endotracheal suctioning in acute respiratory distress syndrome Critical Care 2003 7 55 62 10.1186/cc1844 12617741 PMC154111

[B14] Draper WB Whitehead RW Diffusion respiration in the dog anesthetized by pentothal sodium Anesthesiology 1944 5 262 273 10.1097/00000542-194405000-00004

[B15] Enghoff H son Holmdahl M Risholm L Diffusion respiration in man Nature 1951 168 830 10.1038/168830a0 14890767

[B16] Holmdahl MH Pulmonary uptake of oxygen, acid base metabolism and circulation during prolonged apnoea Acta Chir Scand Suppl 1956 212 1 128 13326155

[B17] Frumin MJ Epstein RM Cohen G Apneic oxygenation in man Anesthesiology 1959 20 789 798 10.1097/00000542-195911000-00007 13825447

[B18] Teller LE Alexander CM Frumin MJ Gross JB Pharyngeal insufflation of oxygen prevents arterial desaturation during apnea Anesthesiology 1988 69 980 982 10.1097/00000542-198812000-00035 3195773

[B19] Taha SK Siddik-Sayyid SM El-Khatib MF Dagher CM Hakki MA Baraka AS Nasopharyngeal oxygen insufflation following pre-oxygenation using the four deep breath technique Anaesthesia 2006 61 427 430 10.1111/j.1365-2044.2006.04610.x 16674614

[B20] Baraka AS Taha SK Siddik-Sayyid SM Kanazi GE El-Khatib MF Dagher CM Chehade J-MA Abdallah FW Hajj RE Supplementation of pre-oxygenation in morbidly obese patients using nasopharyngeal oxygen insufflation Anaesthesia 2007 62 769 773 10.1111/j.1365-2044.2007.05104.x 17635423

[B21] Lumb AB Lumb AB Distribution of pulmonary ventilation and perfusion Nunn's applied respiratory physiology 2005 6 Philadelphia: Elsevier, Butterworth, Heinemann 110 133

[B22] Dunford JV Davis DP Ochs M Doney M Hoyt DB Incidence of transient hypoxia and pulse rate reactivity during paramedic rapid sequence intubation Ann Emerg Med 2003 42 721 728 10.1016/S0196-0644(03)00660-7 14634593

[B23] The American Society of Anesthesiologists task force on management of the difficult airway Practice guidelines for management of the difficult airway Anesthesiology 2003 98 1269 1277 10.1097/00000542-200305000-00032 12717151

[B24] Nielsen ND Andersen G Kjaergaard B Staerkind ME Larsson A Alveolar accumulation/concentration of nitrogen during apneic oxygenation with arteriovenous carbon dioxide removal ASAIO J 2010 56 30 34 10.1097/MAT.0b013e3181c4e935 20038832

[B25] Nielsen ND Kjaergaard B Koefoed-Nielsen J Steensen CO Larsson A Apneic oxygenation combined with extracorporeal arteriovenous carbon dioxide removal provides sufficient gas exchange in experimental lung injury ASAIO J 2008 54 401 405 10.1097/MAT.0b013e31817e2b5f 18645358

[B26] Neumann P Berglund JE Fernández Mondéjar E Magnusson A Hedenstierna G Dynamics of lung collapse and recruitment during prolonged breathing in porcine lung injury J Appl Physiol 1998 85 1533 1543 9760351 10.1152/jappl.1998.85.4.1533

[B27] Rothen HU Sporre B Engberg G Wegenius G Högman M Hedenstierna G Influence of gas composition on recurrence of atelectasis after a reexpansion maneuver during general anesthesia Anesthesiology 1995 82 832 842 10.1097/00000542-199504000-00004 7717553

[B28] Rothen HU Sporre B Engberg G Wegenius G Hedenstierna G Re-expansion of atelectasis during general anaesthesia: a computed tomography study Br J Anaesth 1993 71 788 795 10.1093/bja/71.6.788 8280539

